# Processing Challenges and Opportunities of Camel Dairy Products

**DOI:** 10.1155/2017/9061757

**Published:** 2017-10-03

**Authors:** Tesfemariam Berhe, Eyassu Seifu, Richard Ipsen, Mohamed Y. Kurtu, Egon Bech Hansen

**Affiliations:** ^1^School of Animal and Range Sciences, Haramaya University, P.O. Box 138, Dire Dawa, Ethiopia; ^2^Department of Food Science, University of Copenhagen, Rolighedsvej 30, 1958 Frederiksberg C, Denmark; ^3^Department of Food Science and Technology, Botswana University of Agriculture and Natural Resources, Private Bag 0027, Gaborone, Botswana; ^4^Division for Diet, Disease Prevention and Toxicology, National Food Institute, Technical University of Denmark, 2860 Søborg, Denmark

## Abstract

A review on the challenges and opportunities of processing camel milk into dairy products is provided with an objective of exploring the challenges of processing and assessing the opportunities for developing functional products from camel milk. The gross composition of camel milk is similar to bovine milk. Nonetheless, the relative composition, distribution, and the molecular structure of the milk components are reported to be different. Consequently, manufacturing of camel dairy products such as cheese, yoghurt, or butter using the same technology as for dairy products from bovine milk can result in processing difficulties and products of inferior quality. However, scientific evidence points to the possibility of transforming camel milk into products by optimization of the processing parameters. Additionally, camel milk has traditionally been used for its medicinal values and recent scientific studies confirm that it is a rich source of bioactive, antimicrobial, and antioxidant substances. The current literature concerning product design and functional potential of camel milk is fragmented in terms of time, place, and depth of the research. Therefore, it is essential to understand the fundamental features of camel milk and initiate detailed multidisciplinary research to fully explore and utilize its functional and technological properties.

## 1. Introduction

In many countries, especially the dry zones of Sub-Saharan Africa, camels* (Camelus dromedarius)* play a significant role in the lifestyle of many communities owing to their adaptation to the hostile climatic conditions by providing milk, meat, and transportation. Phenotypic characterization indicated that camels are physiologically, anatomically, and behaviorally adapted to the desert environment. Recent genomic study on the camelid family revealed that the animals carry genetically adapted and evolved genes for desert adaptation such as fat metabolism, osmoregulation, heat stress response, ultraviolet radiation, and choking dust [1]. The genus* Camelus* consists of two different species that live in vast pastoral areas:* Camelus dromedarius*, the one-humped camel, which mainly lives in the desert areas of Africa/Middle East, and* Camelus bactrianus*, the two-humped camel that mainly lives in the cooler dry areas of Asia. More than half of the world's 28 million camel population (old and new world camel) are found in the East African countries, namely, Somalia, Sudan, Ethiopia, and Kenya [2]. Nowadays, the camel is becoming the subject of increasing scientific and commercial interest since climate change is already influencing traditional cattle productivity and the camel is an exceptional animal capable of surviving in the hostile climatic conditions.

Camel milk is composed of lactose, fat, and protein in roughly the same proportion as bovine milk (Table 1). However, their relative composition, distribution, and the molecular structures of the milk components are different. Proteomic analysis of whey proteins of camel, cow, buffalo, goat, and yak milk has revealed that camel milk whey proteins are hierarchically clustered differently than those from the other species [3].

It is commonly claimed that camel milk is difficult to process into products and is only suitable for drinking as fresh or sour milk. But, currently, the possibility of producing various products from camel milk including soft cheese [4, 5], yoghurt [6], and butter [7, 8] has been reported. Also, camel milk has traditionally been recognized for its medicinal values and experimental results indicate that camel milk has antiallergic, antimicrobial, and antidiabetic properties [9, 10]. The objective of this review is to summarize the processing technologies and functional potential of camel milk. Comparison is made with bovine milk, unless otherwise mentioned.

## 2. The Challenges of Processing Camel Milk

The mean gross chemical composition of camel milk is 3.5% fat, 3.1% protein, 4.4% lactose, 0.8% ash, and 12% total solids, which is comparable to bovine milk (Table 1). However, their relative composition, distribution, and the molecular structures of the milk proteins (Tables 2 and 3) and fatty acids (Table 4) are reported to be different.

### 2.1. Processing of Cheese

Processing camel milk into cheese is difficult and has even been considered as impossible [11]. The relative distribution and amino-acid composition of camel milk caseins are different from bovine milk. Camel milk casein has high beta casein (*β*-CN) (65% versus 39%), low alpha S_1_-casein (*α*_s1_-CN) (22% versus 38%), and low kappa casein (*κ*-CN) (3.5% versus 13%) as compared to bovine milk caseins (Table 2). Moreover, the camel milk caseins have low homology to bovine milk caseins, being 39% for *α*_s1_-CN, 64% for *β*-CN, 56% for *α*_s2_-CN, and 56% for *κ*-CN [12]. The chymosin cleavage site of camel milk k-CN was found at the Phe^97^–Ile^98^ amino-acid sequence site, whereas the hydrolysis site in bovine milk is Phe^105^–Met^106^ [12]. Thus, the amount of *κ*-CN in camel milk is relatively small and coagulation of milk in cheese making is typically achieved by enzymatic hydrolysis of *κ*-CN at the surface of casein micelles.

Alpha-lactalbumin (*α*-LA) is the major protein in the camel and human milk whey protein group and *β*-lactoglobulin (*β*-LG) is absent from camel and human milk (Table 3). Camel milk has been reported to contain higher whey protein to casein ration compared to bovine milk which is responsible for a soft and easily digestible curd in the gastrointestinal tract [13]. Camel milk casein has large micelle size with an average diameter of 380 versus 150, 260, and 180 nm compared to bovine, caprine, and ovine milk, respectively [14]. Smaller casein micelles have been reported to improve the gelation properties of bovine milk [15]. Thus, the lower amount of k-CN, the high ratio of whey protein to casein, and the larger micelle size in camel milk are reported reasons for the difficulty of cheese making. These properties result in formation of a less firm coagulum and lower yield during cheese processing. Such low efficiency of cheese processing trials is reported by Bornaz et al. [14] and Konuspayeva et al. [16].

### 2.2. Processing of Butter

The fat content of camel milk ranges from 1.2 to 6.4% [31], which is comparable to that of bovine milk. Nevertheless, butter is not a traditional product made from camel milk and is difficult to produce by using the same technology of production as for butter from bovine milk. The somewhat higher melting point [7, 8] of camel milk fat (41–43°C) makes it difficult to churn the cream at temperatures 10–14°C, which is the optimum churning temperature for bovine milk. Processing of camel milk into butter is also difficult because camel milk shows little tendency to cream up due to deficiency of the protein agglutinin, small fat globule size, and a thicker fat globular membrane [32]. Camel milk is reported to have a higher proportion of long chain fatty acids and a lower amount of short chain fatty acids (Table 4). The high melting point of camel milk butter can be attributed to the high proportion of long chain fatty acids in the fatty acid profile.

However, butter can be made from camel milk under optimum conditions of churning temperature and agitation method. Berhe et al. [7] reported that vigorous shaking of fermented camel milk in a vertical direction instead of the traditional back- and fro-agitation method at a relatively high churning temperature (22-23°C) was able to extract butter from camel milk with a fat recovery efficiency of 80%. This method exerts more force to rupture the fat globule membrane and allow the globules to adhere to one another. Farah et al. [8] also reported that camel milk butter was made at churning temperatures between 15 and 36°C. According to Farah et al. [8], the highest butter fat recovery of 85% was obtained at a churning temperature of 25°C. Camel milk butter is prominently white with a more viscous consistency compared to bovine milk butter. It is reported that pastoralists in the Sahara region produce small amounts of butter from camel milk and they usually use it for medicinal purposes [11].

### 2.3. Processing of Yoghurt

Manufacturing of yoghurt or other fermented products from camel milk is reported to be difficult. Dromedary milk coagulum does not have a desirable curd formation and firmness and the curd is instead fragile and heterogeneous and consists of dispersed flakes [33]. The problem with camel milk yoghurt is thus the thin consistency and weak texture of the product. Yoghurt texture is a very important parameter that affects the appearance, mouth feel, and overall acceptability. Camel milk has been reported to be not easily fermentable because of its antibacterial properties mainly due to the presence of protective proteins. However, growth of commercial starter cultures in camel milk has been found to be possible. The acidification rate in camel milk was, however, lower than in bovine milk [34, 35].

Nevertheless, there are reports that indicate the possibility of yoghurt production from camel milk [36, 37]. Hashim et al. [38] have reported that the firmness of camel milk yoghurt could be improved by supplementation of the milk with gelatin, alginate, and calcium. On the other hand, Al-Zoreky and Al-Otaibi [39] reported that supplementation of stabilizers to camel milk could not improve the consistency of camel milk yoghurt. Ibrahim [40] suggested that the use of exopolysaccharide producing starter cultures could improve the texture of camel milk yoghurt better than additives. There are also reports that indicate the weak texture of camel milk yoghurt can be improved by mixing of camel milk with milk of other livestock species such as ovine milk [41]. The weak texture and thin consistency of camel milk yoghurt can be attributed to the compositional properties of the milk such as lack of *β*-LG and lower amount of k-CN [12], high whey protein to casein ratio [13].

## 3. Functional Potential of Camel Dairy Products 

The unique characteristics of camel milk such as its therapeutic potential and absence *β*-LG have made it a focus area of research in the fields of health science and nutrition as an antimicrobial, antidiabetic and antihypertensive supplement. Camel milk is showing encouraging results in the treatment of autism, cancer, diabetes, and hepatitis and it is safe for children with bovine milk allergy [42–44].

Camel milk has high vitamin C and high mineral contents (sodium, potassium, iron, copper, zinc, and magnesium) and can be good nutritional source for the people living in the arid zones [17, 45].

### 3.1. Therapeutic Properties of Camel Milk

Camel milk has been indicated as safe and efficient in improving long-term glycemic control with a significant reduction in the doses of insulin in type 1 diabetic patients [46, 47]. Agrawal et al. [48] reported that the prevalence of diabetes in the camel milk consuming society of Indian Raica communities was zero. Camel milk is reported to contain insulin-like proteins which is characterized by resistance to gastrointestinal proteolysis, mimics insulin interaction with its receptor, and is easy to absorb into calcium and be encapsulated into lipid nanocapsules [49]. Agrawal et al. [50] have reported that the half cysteine rich protein in camel milk [51] is similar to the insulin family proteins and may be attributed for the glycemic control of camel milk for diabetic patients. Abdulrahman et al. [52] demonstrated that an allosteric effect of camel milk on insulin receptor conformation and activation on the intracellular signaling process may be responsible for the antidiabetic properties of camel milk.

Camel milk was reported to have an antimicrobial effect against Gram-positive and Gram-negative bacteria including* Escherichia coli*,* Listeria monocytogenes*,* Staphylococcus aureus,* and* Salmonella typhimurium* [53]. This inhibitory activity was reported to be due to presence of higher amounts of protective proteins in camel milk including lysozyme, lactoferrin, lactoperoxidase, and immunoglobulins [23, 53, 54].

Camel milk is reported to have antiviral properties. Immunoglobulin and lactoferrin isolated from camel milk could inhibit the hepatitis C virus and demonstrated strong signal against its synthetic peptides, while human counterpart failed to do so [55, 56]. Similarly, camel lactoferrin and lactoperoxidase demonstrated higher inhibition against the entry and direct interaction of hepatitis C virus to Huh7.5 (hepatocyte-derived carcinoma) and HepG2 (human hepatoma) cells [55, 57, 58]. Korashy et al. [59] investigated that camel milk significantly inhibited HepG2 and MCF7 (human breast) cells proliferation and showed induction of death receptors in both cell lines and oxidative stress mediated mechanisms. It is reported that camel milk could play an important role in decreasing oxidative stress by alteration of enzymatic and nonenzymatic antioxidant molecules as well as thymus activation-regulated chemokine levels which resulted in the improvement of child autism in children [60, 61].

Bioactive peptides derived from milk proteins are of great scientific interests due to their nutritional, technological, and potential health benefits. Bioactive peptides can be enriched or released from milk proteins by the use of selected starter cultures and enzymes and by manipulation of the manufacturing processes such as nanofiltration and encapsulation [62–64]. Bioactive peptides with various biological activities such as antihypertensive, antimicrobial, immunomodulatory, antioxidative, antithrombotic, and antiulcerogenic activities have been reported from milk of camels and other livestock species [9]. Many diseases such as Alzheimer, diabetes, atherosclerosis, rheumatoid arthritis, and cancer result from uncontrolled oxidative stresses by excess of free radicals and other reactive oxygen species present in cellular organism [65–67]. Thus, bioactive peptides derived from milk proteins used as an antioxidants play significant role by preventing the formation of radicals or by scavenging the radicals [68, 69].

Camel milk is reported to be a rich source of proteins with potential antimicrobial, angiotensin-converting enzyme (ACE) inhibitory, and antioxidative activities [70–72]. Casein peptides derived from camel milk showed higher antioxidant and ACE-inhibitory activities after enzymatic digestion [73–75]. Most ACE-inhibitory and opioid peptide fractions of the fermented milk mainly contained *β*-CN derived peptides as precursor molecules [62]. The relatively higher content of *β*-CN in camel milk may be advantageous from this point of view. Relatively higher digestibility, antimicrobial activity, and antioxidant activity from camel milk *α*-LA and whey protein was reported [76, 77]. Moslehishad et al. [72] reported that higher ACE-inhibitory and antioxidant activity was observed in cultured camel milk than bovine milk as a result of structural differences and the presence of higher proline content in camel milk caseins. Homayouni-Tabrizi et al. [71] identified two novel antioxidant peptides from camel milk proteins using digestive proteases. Thus, the bioactive properties of camel milk may be responsible for the therapeutic properties of the milk.

### 3.2. The Potential of Camel Milk in Infant Formulations

Infants who are allergic to bovine milk proteins suffer a severe immune response when they ingest nonhuman milk and thus many studies have been done to reduce the allergenicity of bovine milk or to find milks that can substitute bovine milk without producing an allergenic response [78–80]. Human milk is naturally designed to be optimal nutrition for the neonate of the same species. Bovine milk is frequently used to supplement or substitute the human milk for children. Unfortunately, hypersensitivity to bovine milk proteins is a major source of food allergy, which affects primarily infants. The majority of children suffering from bovine milk protein allergy synthesize antibodies primarily against the *β*-LG and *α*_s1_-CN [81, 82].

*In vitro *and* in vivo *experiments showed that camel milk is hypoallergenic and a promising substitute for children who are allergic to bovine milk [83]. Variations in the amino-acid compositions between bovine milk and human milk are reported to be the problems in feeding bovine milk-based infant formulation (Table 5). Human milk casein pattern revealed that the dominance of *β*-CN and *α*s_1_-CN is found in small proportion. Kappeler et al. [12] reported high *β*-CN, lower *α*s_1_-CN, and absence of *β*-LG from camel milk. Thus, this tendency indicates the similarity between camel and human milk. Reports indicated that there was no indicator of immunoglobulin E recognition site at the epitopes of camel milk casein and whey proteins indicating the antigenic difference of camel against bovine, goat, and buffalo milk proteins [80, 84]. Restani et al. [84] indicated that the homologies in amino-acid composition or the phylogenetic differences could justify for difference observed in cross-reactivity between camel and the other livestock species. *α*-LA is a major whey protein in camel milk and camel *α*-LA hydrolysates showed higher digestibility and more antioxidative activity than bovine *α*-LA [76]. These results showed that the chemical composition of camel milk varies considerably from bovine milk. However, chemical composition of camel milk shows remarkable similarity to human milk showing its potential to substitute bovine milk in infant formulations.

### 3.3. Storage Stability of Fermented Camel Milk

Fermented milk products are probably developed from the need to extend the shelf life of milk in the absence of cooling facility, their high nutrient contents, and potential health benefits. Traditionally fermented camel milk is the commonly available camel dairy product unlike camel milk cheese, butter, and yoghurt. Fermented camel milk has different names in different parts of the world;* Shubat *in Turkey, Kazakhstan, and Turkmenistan,* Suusac/susa* in Kenya and Somalia,* Gariss* in Sudan, and* Dhanaan* in Ethiopia. Fermented camel milk is reported to remain relatively stable for a longer time at ambient temperature as a result of the antimicrobial properties of the milk.

*Dhanaan* is reported to have higher storage stability. Pastoralists in Eastern Ethiopia responded that the storage stability of* Dhanaan* is long and it can stay several months when using continuous back slopping, that is, inoculating a new batch of milk with part of a previous batch [85]. Similarly, El-Hadi Sulieman et al. [86] reported that the process of removal of the accumulated* garris* and replacement with fresh camel milk continues for months of time. This might be speculated to the inherent antimicrobial properties of the milk. Potential probiotic lactic acid bacteria strains have been isolated from traditional fermented camel milk and their bacteriocin activities showed inhibitory potential against pathogenic microorganisms [87, 88]. In addition to lactic acid bacteria, yeasts play significant role in the fermentation of traditional camel dairy products since they have strong proteolytic and lipolytic enzymes [87].

## 4. Conclusion

The main reason for the difficulty of producing products from camel milk is due to the unique structural and functional properties of the milk components. Hence, manufacturing of traditional dairy products using the same technology as for dairy products from bovine milk resulted in processing difficulties. Compositional analyses showed that camel milk is similar to human milk. This indicates the potential of using camel milk in infant formulations to alleviate bovine milk allergy in children. The rich source of bioactive components of camel milk and its compositional properties could be attributed to the therapeutic potential of the milk. Information about the processing technologies and functional properties of camel milk is limited. Hence, more detailed study and holistic approach are needed to fully utilize its technological and functional potentials.

## Figures and Tables

**Table 1:** Proximate composition of camel milk compared to milk of other species.

Species	Total solids (%)	Fat (%)	Protein (%)	Lactose (%)	Ash (%)
^a^Camel	12.0	3.5	3.1	4.4	0.8
^b^Bovine	12.7	3.7	3.4	4.8	0.7
^b^Caprine	12.3	4.5	2.9	4.1	0.8
^b^Ovine	19.3	7.4	4.5	4.8	1.0
^b^Human	12.2	3.8	1.0	7.0	0.2

*Sources*. ^a^Al haj and Al Kanhal [17]; ^b^Fox et al. [18].

**Table 2:** Casein protein distribution of camel, bovine, and human milk.

	Caseins (% of total caseins)			Amino acid residues		
	Camel	Bovine	Human	Camel	Bovine	Human
*α*_s1_-casein	22.0^a^	38.0^b^	11.8^c^	217^a^	199^b^	170^d^
*α*_s2_-casein	9.5^a^	10.0^b^	^*∗*^Absent^d^	178^a^	207^b^	^*∗*^Absent^d^
*β*-casein	65.0^a^	39.0^b^	64.0^e^	217^a^	209^b^	212^d^
*κ*-casein	3.5^a^	13.0^b^	24.0^e^	162^a^	169^b^	162^d^
Total caseins (g/100 ml milk)	2.4^c^	2.5^c^	0.4^e^			

^*∗*^Absent indicates corresponding coding sequence is absent in genome. *Sources*. ^*∗*^Kappeler et al. [12], ^b^Eigel et al. [19], ^c^El-Agamy [20], ^d^Martin et al. [21], and ^e^Malacarne et al. [22].

**Table 3:** Whey protein distribution of camel, bovine, and human milk.

	Whey proteins (g/L) in milk			Amino acid residues		
	Camel	Bovine	Human	Camel	Bovine	Human
*β*-Lactoglobulin	^*∗*^Absent^f^	1.3^b^	^*∗*^Absent^c^	^*∗*^Absent^f^	162^b^	^*∗*^Absent^cg^
*α*-Lactalbumin	5.0^f^	1.2^bc^	1.8^cg^	123^f^	123^b^	123^d^
Serum albumin	2.4^h^	0.4^bg^	0.5^gc^	—	582^b^	585^e^
Whey acidic protein	0.16^f^	—	—	117^f^	—	—
Lactoferrin	0.22^a^	0.14^b^	1.5^g^	689^a^	700^b^	700^c^
Immunoglobulins	0.73^fh^	0.7^b^	1.2^g^	—	—	—
Total whey protein	9.3^f^	7.3^f^	7.6^g^			

^*∗*^Absent indicates that corresponding coding sequence is absent in genome. *Sources*. ^a^Kappeler et al. [23], ^b^Madureira et al. [24] ^c^Inglingstad et al. [25], ^d^Findlay and Brew [26], ^e^Meloun et al. [27], ^f^El-Agamy [20], ^g^Malacarne et al. [22], and ^h^El-Hatmi et al. [28].

**Table 4:** Fatty acid profile of camel milk compared to bovine and human milk (% fatty acid).

Carbon number	Fatty acid	Camel milk^ab^	Bovine milk^abc^	Human milk^c^
4:0	Butyric (%)	0.8	1.4	0.1
6:0	Caproic (%)	0.4	2.1	0.2
8:0	Caprylic (%)	0.3	1.7	0.3
10:0	Capric (%)	0.4	3.5	2.0
12:0	Lauric (%)	0.7	3.9	6.8
14:0	Myristic (%)	11.0	12.6	10.4
16:0	Palmitic (%)	29.1	29.5	28.1
18:0	Stearic (%)	12.4	13.3	6.9
Monounsaturated				
14:1		0.5	—	—
16:1	Palmitoleic (%)	10.1	1.7	3.5
18:1	Oleic (%)	24.5	26.3	33.6
Polyunsaturated				
18:2	Linoleic (%)	3.1	2.9	6.4
18:3	Linolenic (%)	1.4	1.1	1.7
Unsaturated/saturated		0.7	0.47	0.82
Short chain (C4–C14)		14.6	25.2	19.8
Long chain (C16–C20)		84.5	72.18	80.2

^a^El-Agamy [10], ^b^El-Agamy [20], and ^c^Malacarne et al. [22].

**Table 5:** Amino acid composition of camel milk proteins compared with bovine and human milk proteins (g/100 g protein).

	Camel^a^	Bovine^b^	Human^c^
*Essential*			
Arginine	4.0	3.7	3.3
Histidine	2.7	3.3	2.8
Isoleucine	5.1	4.9	3.7
Leucine	9.7	9.3	9.5
Lysine	7.2	8.1	10.1
Methionine	3.2	2.5	1.7
Phenylalanine	5.0	4.2	3.9
Threonine	5.7	7.3	8.3
Tryptophan	1.2	1.4	0.5
Valine	6.7	7.6	8.2
*Nonessential *			
Alanine	3.0	4.0	4.2
Aspartic	7.0	7.0	6.7
Cysteine	1.2	0.9	1.0
Glycine	1.5	2.5	2.1
Glutamic	21.7	18.6	16.8
Proline	12.0	9.9	10.6
Serine	5.2	6.2	4.1
Tyrosine	4.6	4.6	2.9

*Sources*. ^a^Mehaia and Al-Kahnal [29], ^bc^El-Agamy et al. [30].
